# Relationship Between Pre- and Post-exercise Body Mass Changes and Pre-exercise Urine Color in Female Athletes

**DOI:** 10.3389/fspor.2022.791699

**Published:** 2022-03-22

**Authors:** Rebecca M. Lopez, Dallin C. Lund, Amanda J. Tritsch, Victoria Liebl

**Affiliations:** Department of Orthopedics and Sports Medicine, School of Physical Therapy and Rehabilitation Sciences, Morsani College of Medicine, University of South Florida, Tampa, FL, United States

**Keywords:** hypohydration, women, body mass changes, weight changes, hydration

## Abstract

Although studies use body mass changes or urine color to measure hydration status, the purpose of this study was to examine the relationship between pre-practice urine color and exercise body mass changes in female tackle football players. Twenty-six female American football players (Age: 29.9 ± 7.3 years; Height: 165.2 ± 2.6 cm; Weight: 83.8 ± 24.4 kg) volunteered. Fluid consumptions (FC) was measured during tackle football practices, while urine color (U_col_), and percent body mass loss (%BML) were taken before and after practices. Subjects were grouped by %BML: lost mass (LM), gained mass (GM), or no change (NC). A one-way ANOVA compared groups on U_col_ and FC. There were differences across groups for pre-practice U_col_ (*P* < 0.01) and FC (*P* < 0.01). GM had a higher pre-practice U_col_ than LM (*P* < 0.01) and NC (*P* < 0.05) and consumed more fluid than LM (*P* < 0.01) and NC (*P* < 0.05). A stepwise linear regression examined the extent that U_col_ and FC were related to %BML. When predicting BML, FC accounted for 45% of variance (*P* < 0.01). The addition of pre-practice U_col_ increased predicted variance explained (*R*^2^ change= 2.5%, *P* = 0032). Subjects who gained mass during practice arrived with elevated urine color (U_col_ 5 ± 2), while those who lost mass arrived with pale urine color (U_col_ 3 ± 2). Findings indicate those who arrived with an elevated urine color attempted to improve hydration status by consuming more fluid and gaining body mass during exercise.

## Introduction

An exercising individual's hydration status can impact a person's health, exercise capacity and athletic performance (Maughan and Shirreffs, 2010a,b). Although there is no gold standard for hydration assessment in field settings, various methods of determining hydration status of athletes during exercise periods have been examined (Armstrong et al., 1994; Shirreffs, 2000; Armstrong, 2007; Maughan and Shirreffs, 2008; Cheuvront et al., 2010). Studies have shown several benefits to being adequately hydrated during exercise (Convertino et al., 1996; Armstrong et al., 1998; American College of Sports et al., 2007; Bandelow et al., 2010; Maughan and Shirreffs, 2010b; Ungaro et al., 2015), while body mass losses of >2% of body weight are often associated with an increased risk of exertional heat illness and decrements in performance (Maughan et al., 2007; McDermott et al., 2017). Gains in body mass are often discouraged due to the risks associated with hyponatremia. Therefore, being able to monitor and quickly assess an athlete's level of hydration is beneficial. Hydration research with female American football players is also very limited.

Measurement of hydration status should be used as a tool to advance athletic performance and potentially decrease injury (Oppliger and Bartok, 2002). Physiologically, hydration plays important roles with temperature regulation, metabolism, transport of substrates across cellular membranes, biochemical reactions, and circulatory function (Armstrong, 2007). Laboratory studies have highlighted the use of various hydration measures, including plasma osmolality, urine osmolality, urine color (U_col_) and body mass changes, when compared to a 3-day baseline body mass measure (Cheuvront et al., 2004, 2010; Armstrong, 2007). Other available methods include hemoglobin testing, hematocrit, urine specific gravity, blood pressure in response to position changes, pulse rate, urinary volume, protein content in urine and in blood, and other blood indices (Shirreffs, 2000). The problem with most of these methods is the availability to test in the field in a manner that a clinician would be able to utilize immediate results for clinical decision making. Although urine specific gravity is easy to measure, using this measure pre-practice may be limited by the time it would take to record this measure for an entire team.

Urine color and body mass changes are some of the easiest most accessible ways to assess hydration in the field setting, and both of these measures have been used individually to assess hydration status when other measurements have not been feasible or practical (Armstrong et al., 1994). However, some suggest using the first void of urine in the morning or comparing body mass changes to the average of 3 consecutive daily body mass measures (Cheuvront et al., 2004; McDermott et al., 2017). Serial body mass measures and collecting athletes' first urine specimens at their homes are not always feasible in the field setting, but, it is important to note the limitations with an on the spot urine color measurement to assess hydration status. The body mass of an athlete is believed to be within 1% of their well-hydrated baseline when urine color is a very pale yellow color under conditions of acute dehydration due to exercise in a hot environment (McKenzie et al., 2015). The physiological impacts of inadequate hydration on athlete health and performance highlight the need for clinicians to have accurate and precise assessment techniques in the athletic setting (Stover et al., 2006; Armstrong, 2007; Munoz and Wininger, 2019). Recent fluid replacement recommendations advise against using only one hydration measure, such as body mass measurements, to determine an individual's hydration status (McDermott et al., 2017). Therefore, combining multiple hydration measures in the field setting may be the best strategy when attempting to assess athlete hydration status before, during and after exercise.

Clinicians (i.e., athletic trainers) can use body mass loss and urine color measurements to educate the athlete on how they can ensure they are euhydrated at the start of exercise to broadly guide fluid consumption during exercise. Voluntary drinking sometimes results in over hydration but can also result in dehydration, even when athletes have access to fluids (Szlyk et al., 1987; Rivera-Brown et al., 1999, 2008; Passe et al., 2007; Wilk et al., 2007; Kavouras, 2019). However, when educating athletes about hydration, it is imperative for athletes to have as much information about their fluid replacement, fluid needs, and hydration status. To date, no study has examined the relationship and impact between pre-exercise urine color and body mass change during exercise. Basing hydration recommendations and clinical decisions on only one of these measures without the other omits an important piece of the puzzle; and that is that a person's hydration status at the start of exercise will surely impact their hydration practices during exercise. Examining this relationship between pre-practice urine color and body mass changes may provide clinicians with a practical and valid means of making a sounder judgement regarding athletes' hydration status and behaviors during exercise.

The purpose of this study was to determine if body mass changes from pre- to post-exercise were related to pre-practice urinary hydration measures during outdoor tackle football practices. We hypothesized that participants whose urinary hydration measures were reflective of hypohydration (i.e., higher urine color) at the start of exercise may gain more body mass by compensating with fluid consumption due to starting exercise in a hypohydrated state. Similarly, we hypothesized that participants whose urinary measures were indicative of being euhydrated prior to exercise may lose body mass from pre- to post-exercise.

## Materials and Methods

### Participants

Subjects from this study were female professional football players (*n* = 26; Age: 29.9 ± 7.3 years, range: 22–48 years; Height: 165.2 ± 2.6 cm; Mass: 83.8 ± 24.4 kg; Body Mass Index: 30.8 ± 9.4; BSA: 1.9 ± 0.2). Exclusion criteria included any injury or condition that would preclude any football player from participating in football practices. This study was approved by the Institutional Review Board, and all participants read and signed an informed consent form. This was a convenience sample of participants from the same team.

### Protocol

This study took place during evening practices during the month of May in the southeastern United States (Wet bulb globe temperature: 22.0 ± 1.8°C). This is the start of the official season for women's tackle football. Data for each subject was recorded over the course of between 4 and 8 practices across two seasons. Because this study ranged across two seasons, not all participants were in attendance for every data collection day. Each football practice lasted 2 h, and participants were wearing full American football gear (helmets, shoulder pads, padding pants, socks, cleats). When participants arrived at practice, they provided pre-practice urine samples for urine color in a standard, clear specimen cup, and semi-nude (shorts and sport bra) body mass measures (Tanita BWB-800S; Tanita Corporation) were then recorded (Armstrong, 2007). An individualized water bottle was marked so that each athlete's fluid consumed was measured for each practice by weighing each bottle (Tanita, KD-404; Tanita Corporation) before and after fluid was consumed (Lopez et al., 2019). Individualized water bottles were moved around during practices so that participants could access their bottle and drink water *ad libitum*. Each individual participant's fluid consumed was measured and recorded throughout practices as needed and at the end of each practice. Post-practice measures were the same as pre-practice measures; however, post-practice body mass was measured first, followed by the post-practice urine sample. Pre- and post-body mass measurements were used to calculate body mass loss (pre-body mass—post-body mass/pre-exercise body mass) x100 (Armstrong, 2005). Urine color was measured by the same 2 testers for all practices by Armstong's original urine color chart (Armstrong, 2000). The same data collection procedures were followed for all data collection days (Lopez et al., 2019).

### Statistical Analysis

Descriptive data (mean ± SD) were calculated for all variables. Due to the within-subject repeated nature of the data, a multi-level model was used to examine the extent that the measure of hydration (pre-practice urine color) and fluid consumed were related to body mass loss. The grouping variable was individual subject, with BML as the dependent variable, and fluid consumed and urine color as co-variates.

To further investigate the potential differences in hydration and fluid consumption when one gained, lost, or maintained body mass throughout an individual practice, data were grouped by body mass loss (LM), body mass gain (GM), and no body mass change (NC). An ANOVA was then used to compare the groups on fluid consumed, pre-practice urine color, and post-practice urine color. Given the within subject design that was not dependent on practices in this global assessment, all data were collapsed across days. While the ANOVA cannot account for the potential violation of independence due to collapsing across days, we wished to demonstrate in absolute numbers how BML and fluid consumed varied by pre-practice urine color on a given practice in way the linear multi-level modeling does not demonstrate. The significant α level was set at *P* < 0.05. SPSS 28 for Mac was used for all analyses.

## Results

### Relationship Between Hydration and Body Mass Loss

**Figure 1:**
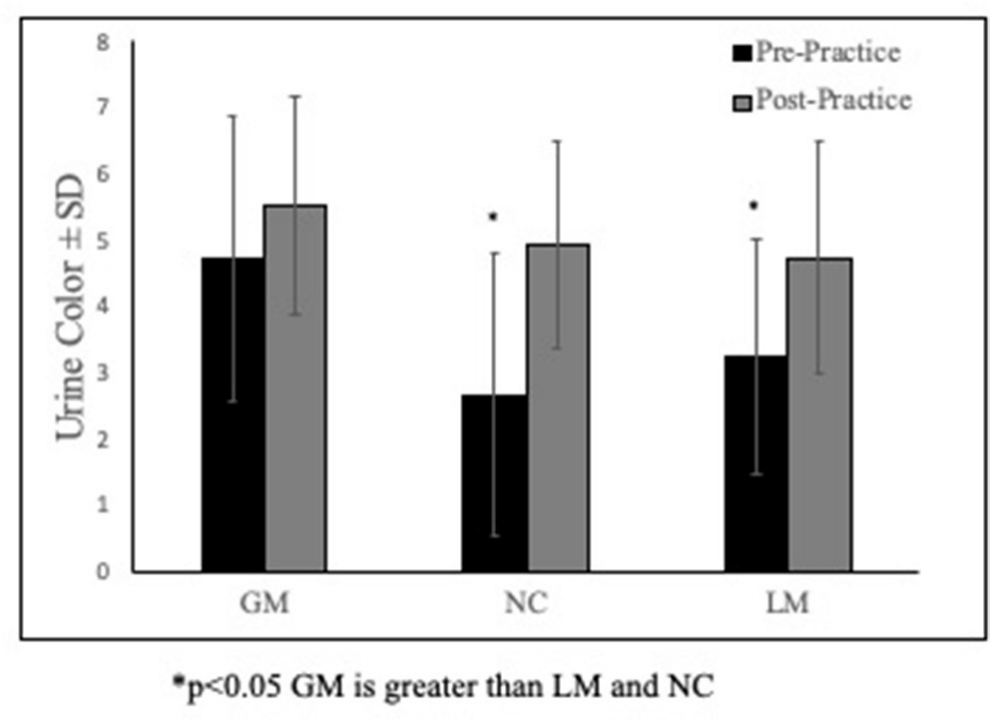
Group differences in pre- & post-practice urine color. *p <0.05 GM is greater than LM and NC.

The model can be found in Table 1. Figure 1 demonstrates the relationship between pre-practice urine color and percent BML. The estimates indicate that for every 1 mL of fluid consumed (β = 0.001), there was a percent BML change of 0.001 and for every unit change on the urine color scale (β = 0.031) there was a percent BML change of 0.031. In more clinically relevant terms, for every liter of fluid consumed, participants gained 0.1% mass, or consuming more water resulted in losing less body mass. Lastly, greater pre-practice urine color predicted losing less body mass.

**Table 1:** Multi-level model summary.

**Parameter**	**Estimate (coefficient)**	***P*** **-value**
Constant	−0.981	<0.001
Fluid consumed	0.001	<0.001
Urine color	0.031	0.188

Overall Mean, SD, and range for BML and fluid consumed are listed in Table 2. Of the 104 total data points, 21 were categorized as GM, 71 as LM, and 12 as NC.

**Table 2:** Overall and group means ± SD.

	**BML %**	**Fluid consumed (mL)**
Gained mass (*n* = 21)	0.5 (0.4)	1256.6 (536.0)
Lost mass (*n* = 71)	−0.6 (0.3)	620.0 (302.4)^*^
No mass change (*n* = 12)	0.0 (0.0)	908.1 (365.1)^*^^†^
Overall (*n* = 104)	−0.3 (0.5)	781.8 (445.0)

* *P < 0.05 GM was greater than LM and NC groups*.

† *P < 0.05 NC was greater than the LM group*.

*BML%, percent body mass loss*.

### Pre-practice Hydration and Body Mass Loss

There were differences across groups for pre-practice urine color (*P* < 0.01) and fluid consumed (*P* < 0.01). Specifically, the group that gained mass throughout practice (GM) had a higher pre-practice urine color than the group who lost mass, or LM (*P* < 0.01) and the no mass change (*P* < 0.05) groups (Figure 1). The GM group also consumed more fluid than LM (*P* < 0.01) and the NC (*P* < 0.05) groups (Table 2). The NC group also consumed more fluid than LM (*P* < 0.01).

## Discussion

**Figure 2:**
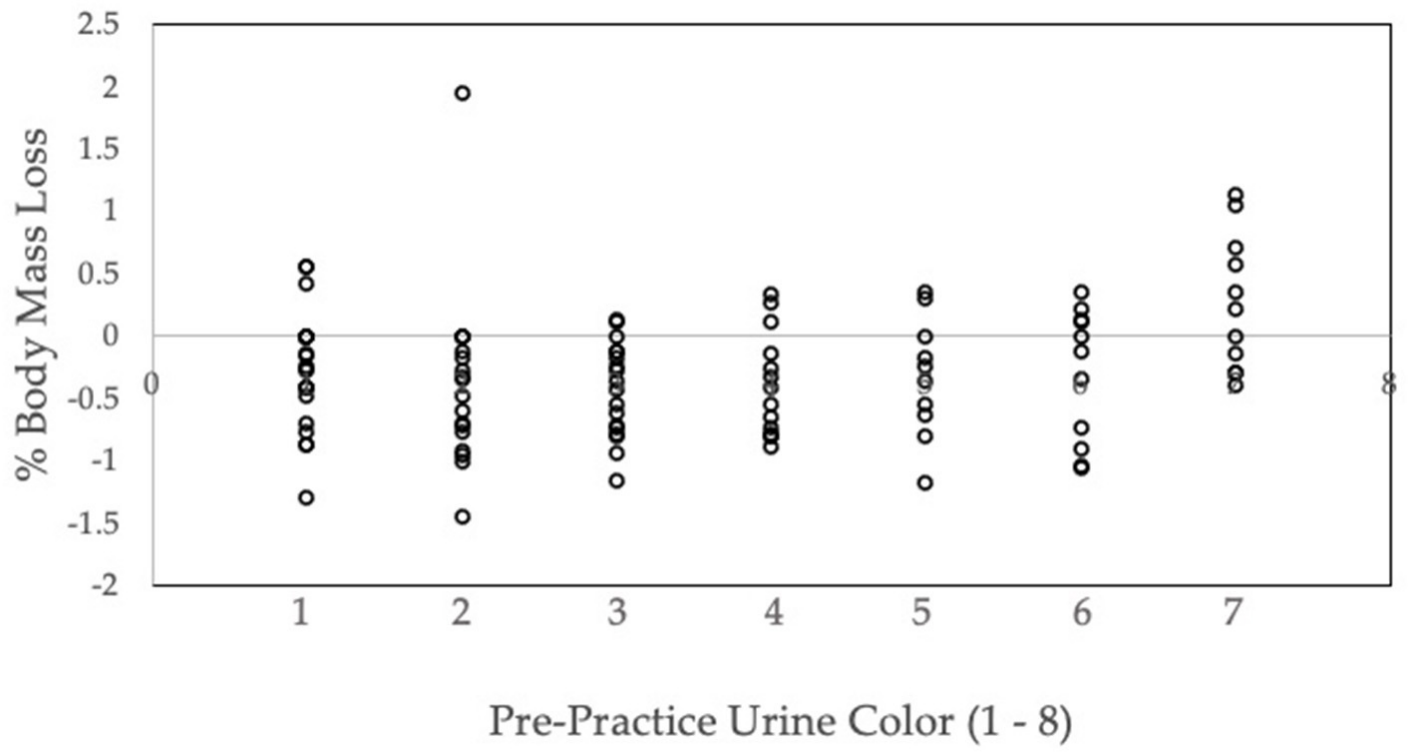
Pre-practice urine color and percent body mass loss. Individual data for percent body mass loss (% BML) and pre-practice urine color.

To our knowledge no study has specifically examined the relationship between pre-practice urinary hydration measures and body mass changes during female football practices in the field setting. Furthermore, the present study sought to examine whether those athletes that arrived in a hypohydrated state compensated for this during exercise by consuming more water. When looking at pre-practice urinary measures, we found that subjects who gained mass pre- to post-exercise arrived at practice in a hypohydrated state with an average urine color of 5 (Figures 1, 2). Furthermore, those who lost mass from pre- to post-practice arrived in a euhydrated state with a urine color of 3. Collectively, this may indicate that the group that did not change mass best matched their sweat rate throughout practice. From a clinical standpoint, these findings suggest that the group that gained mass during exercise (which is often discouraged) may have been consuming fluid not only to match their sweat rates but also to make up for their hypohydrated state that they reported to practice with. Many fluid replacement recommendations suggest exercising individuals should never gain mass during the course of an exercise bout (Hew-Butler et al., 2015; McDermott et al., 2017). However, knowing the individual's hydration status at the start of exercise is imperative to make recommendations that would prevent both exertional hyponatremia as well as extreme hypohydration. In the present study, those that arrived with a higher urine color also consumed the most fluid (Table 2); thus, these findings demonstrate how reliance on only one hydration measure in the field setting may be misleading.

The direct relationships between hydration and BML revealed that for every liter of fluid consumed each participant was predicted to gain 0.1% body mass, and that consuming more water resulted in less predicted body mass loss. Additionally, greater pre-practice urine color predicted losing less body mass (Figure 1 and Table 2). Our findings support recommendations that hydration and rehydration with competitive sports should take an individualized approach and further supports recommendations to use more than one hydration measure to best capture an individual's hydration status in the field setting (Maughan and Shirreffs, 2010b; McDermott et al., 2017). These findings may also indicate that some athletes are able to compensate for high pre-practice urine colors by increasing fluid consumption during practices.

Using body mass to assess hydration can be one of the most common ways to measure hydration status, particularly in individuals that are euhydrated at the start of exercise; however, having a euhydrated pre-exercise body mass helps better quantify any changes in body mass during exercise. Body mass changes can be equated with losses of body water (1 kg = 1 L) with very minimal error during short duration exercise (Cheuvront et al., 2015). Although 3 consecutive body mass measurements were not feasible in this study, we paired body mass measures and U_col_ to better depict the hydration status of these athletes in a field setting. Athletes are often educated about body mass changes and encouraged to avoid both fluid losses >2% of their body mass as well as to prevent body mass gains during exercise.

Several variables can impact the hydration education individuals receive regarding their post-exercise rehydration needs. Rehydration post-exercise is going to be dependent on the duration and type of exercise, dietary needs of the athlete, and individual sweat rate. Hydration education on post-exercise rehydration is also necessary to ensure fluids are adequately replaced prior to the next exercise bout (Maughan et al., 1997; Armstrong et al., 1998; Maughan and Shirreffs, 2010b; Park et al., 2012; Atkins et al., 2021). Current guidelines suggest either replacing fluids to match sweat losses or replacing 150% of fluid losses when there is a short recovery period (i.e., 4 h) (Shirreffs et al., 2007; McDermott et al., 2017). However, these recommendations may not be sufficient for the following reasons (Shirreffs et al., 2007). In the present study, using body mass losses alone to provide fluid replacement and hydration education to the participants would have resulted in recommendations based on incomplete or inaccurate information. For example, in the athletic setting, if only body mass changes from pre- to post-exercise are measured, a gain in body mass during exercise may lead a clinician to advise an athlete to decrease fluid consumption to prevent hyponatremia. Knowing that an athlete began exercise in a hypohydrated state would considerably change the education a clinician delivers to that same athlete. In that case, a pre-exercise urine color indicative of hypohydration (5 or higher) should warrant education stressing the importance of consuming sufficient fluids before exercise. As demonstrated by the individual data presented in Figure 2, some participants' pre-practice urine was at a 5 or higher and still lost body mass while others gained mass due to fluid replacement. Therefore, we recommend that clinician's test hydration status via both monitoring both urine color before exercise along with body mass changes during exercise (Cheuvront et al., 2015).

In laboratory studies examining body mass changes during exercise, study subjects are often instructed to arrive to the laboratory in a euhydrated state. However, field studies have demonstrated (*via* various pre-exercise urinary hydration measures) that athletes often arrive to practice in a hypohydrated state (Decher et al., 2008; McDermott et al., 2009; Silva et al., 2010; Yeargin et al., 2010; Arnaoutis et al., 2013, 2015). In the present study, by examining both the pre-exercise hydration status (i.e., urine color) and body mass changes, we were able to determine that body mass gains during exercise were due to the athletes trying to compensate for being hypohydrated at the start of practice or if they were truly hyperhydrating. It is unclear whether the athletes knew they were hypohydrated and intentionally tried to consume more fluid or if thirst or other factors impacted this behavior. The latest hydration and fluid replacement recommendations include using multiple hydration measures rather than using one measure on its own. However, there is a lack of studies examining the relationship between mass gained during exercise and pre-exercise hydration measures.

This study was not without limitations. Data collection for this study took place across two separate seasons. Although not ideal, this was due to the timing of the season and the nature of women's tackle football having fewer participants compared to traditional high school and collegiate football teams. As such, these participants attend practice outside of their work and family commitments. As compared to a controlled laboratory study, the field study setting did not allow for the control for dietary or physical activity outside of the practices, nor were we able to control for hormonal status.

Another limitation was the use of a spot urine measurement for determining hydration status, which has been discouraged (Cheuvront et al., 2015). The first void of the day is often recommended for the most accurate urinary hydration measures, however, this is not always possible depending on when a team is scheduled to practice. The practices in the present study were in the evenings; therefore, the first morning void would not have given an accurate reading of their pre-practice hydration status. A recent study indicated that weigh charts are often not used or used incorrectly (Eith et al., 2020); therefore, we aimed to determine how to best use practical measures to guide fluid replacement recommendations for athletes. We also understand that urine color may be affected by dietary factors, and we did not control for dietary or physical activity prior to practices; however, urine color appears to be the most feasible and practical urinary hydration measure in the field setting (Armstrong et al., 1994, 1998, 2010). While not a gold standard, urine color is strongly correlated with urine specific gravity and urine osmolality and is considered an acceptable hydration measure in the field setting (Armstrong et al., 1994, 1998). The clear specimen cup used for the urine sample may have been a limitation if the participant observed their urine color. It is not entirely clear whether the participants' hydration behavior was affected by knowledge of their own fluid needs, sweat rate or hydration status at the end of practice. Future research should examine the factors that would affect hydration behavior during exercise.

## Conclusions

The results from the present study highlight the need for clinicians to use multiple, easy and non-invasive hydration measures to best determine hydration status in athletes. Our findings support the recommendation that combining two practical and feasible measures in the field setting may be more accurate than using only one of these measures. Clinicians should note that active individuals that begin exercise in a hypohydrated state may consume more fluid, and therefore, gain mass during exercise to prevent further dehydration during exercise. Conversely, athletes that are euhydrated at the start of exercise are more likely to drink less fluid and therefore have greater fluid losses. We demonstrated that body mass changes taken pre- and post-practice can be a useful diagnostic tool to assess hypohydration in female football players, particularly when used in conjunction with pre-exercise urine color. Future research is needed to further examine the accuracy of these hydration methods on different athletes, at different levels, and playing different sports. Athletic trainers and other healthcare providers should use multiple hydration assessment measures (i.e., urine color and body mass together) in order to provide accurate, individualized hydration recommendations to their patients.

## Data Availability Statement

The raw data supporting the conclusions of this article will be made available by the authors, without undue reservation.

## Ethics Statement

The studies involving human participants were reviewed and approved by the Institutional Review Board of the University of South Florida. The patients/participants provided their written informed consent to participate in this study.

## Author Contributions

RL, DL, and AT: conceptualization. RL and AT: methodology, formal analysis, data curation, visualization, and supervision. AT: software. RL, DL, AT, and VL: validation, writing—original draft preparation, and writing—review and editing. RL: investigation, resources, project administration, and funding acquisition. All authors have read and agreed to the published version of the manuscript.

## Funding

This research was funded in part by the University of South Florida New Investigator Grant, # 18325-0083616.

## Conflict of Interest

The authors declare that the research was conducted in the absence of any commercial or financial relationships that could be construed as a potential conflict of interest.

## Publisher's Note

All claims expressed in this article are solely those of the authors and do not necessarily represent those of their affiliated organizations, or those of the publisher, the editors and the reviewers. Any product that may be evaluated in this article, or claim that may be made by its manufacturer, is not guaranteed or endorsed by the publisher.
